# Special Hospitals

**Published:** 1909-04-10

**Authors:** 


### HOSPITAL ADMINISTRATION.

### CONSTRUCTION AND ECONOMICS.

### SPECIAL HOSPITALS.

We have lately received an interesting, well-written monograph* by a man who has devoted the best years of his life to the cause of special hospitals. It is not too much to say that the continuous success of the Central London Hospital for Diseases of the Throat, Nose and Ear, in Gray's Inn Road, is largely due to the devotion of the secretary to its interests. The book contains a few interesting plates dealing with ancient medicine, and a reasonably complete chronology of the origin and development of modern special hospitals, in the course of which the names of most of the men who have been instrumental in establishing these institutions finds a place. Due tribute is paid to medical women, and their enterprise and influence in the hospital world is recorded. Thanks to Mr. Kershaw's zeal and energy, the Central London Throat and Ear Hospital has been conducted with great efficiency and economy, and has always deserved credit for the care taken by the management to prevent hospital abuse. There is a chapter on special hospitals and their relation to medical education, in the course of which it is shown that 676 medical practitioners visited the eye hospitals, and a similar number the ear and throat hospitals, to witness their practice in 1907, in addition to 294 post-graduate students. Seven hundred and sixty medical practitioners and post-graduates visited the special hospitals for diseases of the skin in the same year with the same object. The number of medical practitioners in the same year who visited the following groups of special hospitals were:—

| | |
|---|---|
| Hospitals for women ... ... ... | 574 |
| Hospitals for paralysis ... ... ... | 725 |
| Hospitals for children ... ... ... | 420 |

It is a remarkable fact that 1,383 medical practitioners are stated to have visited St. Peter's Hospital for Stone in 1907. In the whole 5,735 medical visitors and 547 students attended the practice of 22 special hospitals in London in 1907.

There is a chapter on special hospitals in relation to economy, in the course of which Mr. Kershaw contends that if the average cost per patient is taken at all the general hospitals and all the specials, "the economy certainly rests with the latter." Mr. Kershaw states that the average cost of each in-patient per week at 17 general hospitals is £1 17s. 4d., and the average cost of each out-patient's attendance is 7¼d. The average cost of each in-patient per week and of each out-patient's attendance at the following groups of special hospitals is stated to be as follows:—

| | Average weekly cost of each in-patient. £ s. d. | Average cost of each out-patient's attendance. d. |
|---|---|---|
| Women's Hospitals ... ... | 1 17 9 | 9¾ |
| Women and children ... ... | 1 9 7 | 6¼ |
| Consumption ... ... ... | 1 10 3¾ | 10¾ |
| Ophthalmic hospitals ... ... | 1 3 10 | 5 |
| Throat and ear hospitals ... | 1 14 5¾ | 8¼ |
| Paralysis ... ... ... | 1 13 8 | 9¾ |
| Miscellaneous special hospitals | 2 2 9 | 10 |

Taking the whole 15 general hospitals and 31 special hospitals, Mr. Kershaw's figures are as follows:—

| | General Hospitals. £ s. d. | Special Hospitals. £ s. d. |
|---|---|---|
| Average cost of each in-patient per week ... ... ... | 1 17 4 | 1 13 0 |
| Average cost of each out-patient's attendance ... | 7¼ | 8½ |

Mr. Kershaw gives the following interesting figures concerning the Central London Throat and Ear Hospital for the year 1908:—Of 10,481 new out-patients and 706 in-patients, 3,067 out-patients and 392 in-patients were sent to the hospital by medical practitioners. The out-patients made 47,406 attendances, of which 29,450 were paid for by the patients' contributions, varying from 6d. to 15s.; 16,205 paid 1s., 7,538 paid 2s., 1,984 contributed 6d., 1,008 1s. 6d., 1,094 3s., 700 4s., 220 5s., 14 10s., and 13 amounts varying from 10s. to 15s. each. Of the remaining attendances 16,296 were absolutely free, and 1,660 were admitted by subscribers' letters. It may be added that the payments extended for periods varying from a week to a month, but the sixpences and shillings represent a week's treatment, whilst the higher payments were for a more lengthened period. Forty per cent. of the out-patients and 60 per cent. of the in-patients at the hospitals for women were recommended by medical practitioners, whilst 29 per cent. of the out-patients and 55 per cent. of the in-patients at the Central London Throat and Ear Hospital were similarly recommended.

The book concludes with a list of the special hospitals founded in the United Kingdom during the nineteenth century, the date of foundation, the names of the founders and statistics of medical and post-graduate visitors, and of the patients sent to each institution by medical practitioners. This monograph is well printed, its contents are interestingly written and well illustrated, whilst the statistics are clearly given.

* "Special Hospitals: their Origin, Development, and Relationship to Medical Education. Their Economic Aspects and Relative Freedom from Abuse." By Richard Kershaw. (London: Geo. Pulman and Sons, Limited, Thayer Street, W.)

